# Mitochondrial Respiration in Human Colorectal and Breast Cancer Clinical Material Is Regulated Differently

**DOI:** 10.1155/2017/1372640

**Published:** 2017-07-11

**Authors:** Andre Koit, Igor Shevchuk, Lyudmila Ounpuu, Aleksandr Klepinin, Vladimir Chekulayev, Natalja Timohhina, Kersti Tepp, Marju Puurand, Laura Truu, Karoliina Heck, Vahur Valvere, Rita Guzun, Tuuli Kaambre

**Affiliations:** ^1^Laboratory of Bioenergetics, National Institute of Chemical Physics and Biophysics, Tallinn, Estonia; ^2^Oncology and Haematology Clinic at the North Estonia Medical Centre, Tallinn, Estonia; ^3^Laboratory of Fundamental and Applied Bioenergetics, INSERM, University Grenoble Alpes, U1055 Grenoble, France; ^4^School of Natural Sciences and Health, Tallinn University, Tallinn, Estonia

## Abstract

We conducted quantitative cellular respiration analysis on samples taken from human breast cancer (HBC) and human colorectal cancer (HCC) patients. Respiratory capacity is not lost as a result of tumor formation and even though, functionally, complex I in HCC was found to be suppressed, it was not evident on the protein level. Additionally, metabolic control analysis was used to quantify the role of components of mitochondrial interactosome. The main rate-controlling steps in HBC are complex IV and adenine nucleotide transporter, but in HCC, complexes I and III. Our kinetic measurements confirmed previous studies that respiratory chain complexes I and III in HBC and HCC can be assembled into supercomplexes with a possible partial addition from the complex IV pool. Therefore, the kinetic method can be a useful addition in studying supercomplexes in cell lines or human samples. In addition, when results from culture cells were compared to those from clinical samples, clear differences were present, but we also detected two different types of mitochondria within clinical HBC samples, possibly linked to two-compartment metabolism. Taken together, our data show that mitochondrial respiration and regulation of mitochondrial membrane permeability have substantial differences between these two cancer types when compared to each other to their adjacent healthy tissue or to respective cell cultures.

## 1. Introduction

The field of cellular bioenergetics is gaining increased attention and studies performed during the last years have shown that targeting cancer cell energy metabolism may be a new and promising area for selective tumor treatment [1]. The literature describing changes in energy metabolism and mitochondrial function during carcinogenesis is, unfortunately, full of contradictions. Majority of previous studies about the bioenergetics of malignant tumors were performed in vitro on different cell models with the conclusion that cancer cells have increased glucose uptake and, due to mitochondrial damage, it is not metabolized via oxidative phosphorylation (OXPHOS) [2–4]. It is clear that for many tumors, glycolysis is the main energy provider, but in others, OXPHOS is still crucial for survival and progression and produces necessary ATP [1, 5, 6]. Recently, a new concept for tumor metabolism was proposed—metabolic coupling between mitochondria in cancer cells and catabolism in stromal cells—which promotes tumor growth and development of metastases. In other words, tumor cells induce reprogramming in surrounding nontumor cells so that the latter acquire the Warburg phenotype [7] and start producing and exporting the necessary fuels for the anabolic cancer cells (“reverse Warburg”). The cancer cells will then metabolize these fuels via their tricarboxylic acid cycle and OXPHOS [8–10]. Complex interplay between developing cancer cells and host physiology, possibly mediated by “waves” of gene expression in the tumor [11, 12], can only develop in vivo and therefore in vitro studies cannot give conclusive information about the functional activity and capacity of OXPHOS in human samples. In vitro models ignore many factors arising from the tumor microenvironment (TME), which can and will exert significant effects in vivo. TME consists of nonmalignant cells, soluble growth factors, signaling molecules, and extracellular matrix that support tumor progression [13], but high heterogeneity within cancers cell population on top of it contributes to even further complexity in clinical samples [14]. At the same time, the metabolic profiles of tumor cells that are grown in culture have significant variations primarily due to the culture conditions, such as concentrations of glucose, glutamine, and/or fetal serum. Cells grown in glucose-free medium display relatively high rates of oxygen consumption, but cultivation in high-glucose medium increases their glycolytic capacity together with reduced respiratory flux [15–19].

In addition to intercellular differences, there are also intracellular rearrangements resulting from tumor formation. The functional units within cells are often macromolecular complexes rather than single species [20]. In case of OXPHOS, it has been shown that complexes of the respiratory chain can form assemblies—supercomplexes—that lead to kinetic and possibly homeostatic advantages [21]. Therefore, pure genome or transcriptome data are not sufficient for describing the final in situ modifications and the final outcomes of a pathway or cellular processes are defined by actual activities of their separate proteins—or their assemblies—together with the respective regulatory mechanisms. More specifically, previous studies have shown that in cardiac and yeast cells, a large protein supercomplex is centrally positioned in regulation of mitochondrial respiration and mitochondrial energy fluxes. The supercomplex consists of ATP synthasome, mitochondrial creatine kinase (MtCK) or hexokinase (HK), voltage-dependent anion channel (VDAC), and some regulatory proteins expectedly coordinate the selective permeability of it. This complex is known as mitochondrial interactosome (MI) [22], and it is located in the contact sites of outer and inner mitochondrial membranes. This unit also includes supercomplexes formed by the respiratory chain [23, 24]. Changes in the content of ATP synthasome and respiratory chain supercomplexes in pathological conditions are still poorly studied. Inhibiting key respiratory enzymes or avoiding restructuring of mitochondrial supercomplexes in tumors has potential to disrupt disease progression without affecting normal cells, thus, providing a powerful new approach for developing novel therapeutic targets. Specifically, Rohlenova et al. recently demonstrated that breast cancer cells expressing HER2 oncogene develop specific RC supercomplexes which make complex I in these susceptible to treatment with chemically altered tamoxifen called MitoTam [25]. MitoTam is taken to a phase I clinical study [25], and there are other clinical studies undergoing that target OXPHOS in different cancer types (e.g., trial numbers NCT01957735 and NCT02650804). Therefore, despite the assumed glycolytic nature of human tumors, inhibition of oxidative respiration is proving to be a viable therapeutic strategy and further studies are needed to define differences between cancer types but also individual patients in regard to such treatment.

We have previously shown on clinical samples that both human breast cancer (HBC) and human colorectal cancer (HCC) are not purely glycolytic, but these tumors have sustained OXPHOS as a substantial provider of ATP [26–28]. Here, we extend our studies by comparing bioenergetics of HBC and HCC using kinetic methods.

## 2. Materials and Methods

### 2.1. Chemicals

All chemicals were purchased from Sigma-Aldrich (USA) and were of the highest purity available (>98%).

### 2.2. Clinical Materials

The tissue samples were provided by the Oncology and Haematology Clinic at the North Estonia Medical Centre (Tallinn). All the samples were analyzed immediately after surgery. Only primary tumors were examined and information from respective pathology reports was provided by the North Estonia Medical Centre for all the analyzed samples. Informed consent was obtained from all the patients and coded identity protection was applied. All investigations were approved by the Tallinn Medical Research Ethics Committee and were in accordance with the Helsinki Declaration and Convention of the Council of Europe on Human Rights and Biomedicine. The entire group consisted of 34 patients with breast cancer and 55 with colorectal cancer.

### 2.3. Cell Cultures

MDA-MB-231 and MCF-7 cells were grown as adherent monolayers in low glucose (1.0 g/L) Dulbecco's modified Eagle's medium (DMEM) with stable L-glutamine and sodium pyruvate (from Capricorn Scientific GmbH) supplemented with 10% heat-inactivated fetal bovine serum, 10 *μ*g/mL human recombinant Zn insulin, and antibiotics: penicillin (100 U/mL), streptomycin (100 *μ*g/mL), and gentamicin at a final concentration of 50 *μ*g/mL. Cells were grown at 37°C in a humidified incubator containing 5% CO_2_ in air and were subcultured at 2-3-day intervals.

### 2.4. Mitochondrial Respiration in Saponin-Permeabilized Tissue Samples

Numerous studies have demonstrated that isolated mitochondria behave differently from mitochondria in situ [29–32]. We therefore have investigated respiratory activity of tumor and control tissues in situ using the skinned sample technique [26, 28, 29, 33]. This method allows analysis of the function of mitochondria in cells in their natural environment and leaves links between cytoskeletal structures and mitochondrial outer membranes intact [34–37]. Cytochrome c test was used to confirm integrity of the mitochondrial outer membrane (MOM) [22, 26, 28, 33]; mitochondrial inner membrane quality was checked using a carboxyatractyloside (CAT) test as the last procedure in every experiment [22, 26, 28, 33]. Rates of O_2_ consumption were assayed at 25°C using Oxygraph-2k high-resolution respirometer (Oroboros Instruments, Innsbruck, Austria) loaded with pre-equilibrated respiration buffer medium B [26]. Activity of the respiratory chain was measured by substrate-inhibitor titration as described earlier [26, 38]. The solubility of oxygen at 25°C was taken as 240 nM/mL [39]. The solubility of oxygen is much lower at 37 than at 25°C, but also, the skinned samples from malignant clinical material are more stable at 25°C. All rates of respiration (*V*) are expressed in nM O_2_/min per mg dry tissue weight for solid tumors and in nM O_2_/min per million cells for cell cultures.

### 2.5. Metabolic Control Analysis

Metabolic control analysis (MCA) is a method for studying regulatory mechanisms in complex metabolic systems [40–42]. Flux control coefficient (FCC) is defined as the ratio of fractional change in a system variable to fractional change in a biochemical activity that caused the change in the given system [42]. FCC or *C*_*vi*_^*J*^ is the extent to which an enzyme in a pathway controls the flux (*J*); it corresponds to the percentage decrease in flux caused by a 1% decrease in the activity (𝑣_i_) of that enzyme [41, 43]:

(1) $$\begin{array}{c} C_{vi}^{J}=\frac{\left(dJ/{dv}_{i}\right)}{\left(J/v_{i}\right)}=\frac{d\ln J}{d\ln v_{i}}. \end{array}$$

This method shows how the control is shared between the enzymes and the transporters of the pathway and enables to identify the steps that could be modified to achieve successful alteration of the flux or metabolite concentration in the pathway. But it also permits the identification of system components that are crucial in the regulation of energy transfer and regulatory networks [40–42, 44–46].

MCA has previously been applied in our lab to human breast and colorectal cancer skinned samples to determine the FCCs for respiratory chain complexes. The flux was measured as the rate of O_2_ consumption in permeabilized tissues derived from HCC patients when all components of the OXPHOS system were titrated with specific irreversible or pseudoirreversible inhibitors to stepwise decrease selected respiratory chain complex activities according to a previously published method [26, 27, 47, 48].

### 2.6. Western Blot Analysis of the Level of Mitochondrial RC Complexes Expression

Postoperative human tissue samples (70–100 mg) were crushed in liquid nitrogen and homogenized in 20 volumes of RIPA lysis buffer (50 mM Tris-HCl pH 8.0, 150 mM NaCl, 2 mM EDTA, 0.5% sodium deoxycholate, 0.1% SDS, 0.1% Triton X-100, and complete protease inhibitor cocktail (Roche)) by Retsch Mixer Mill at 25 Hz for 2 min. After homogenization, samples were incubated for 30 min on ice and centrifuged at 12,000 rpm for 20 min at 4°C. The proteins in the supernatants were precipitated using acetone/TCA to remove nonprotein contaminants. Briefly, supernatants were mixed with 8 volumes of ice-cold acetone and 1 volume of 100% TCA, kept at −20°C for 1 h and then pelleted at 11500 rpm for 15 min at 4°C. The pellets were washed twice with acetone and resuspended in 1x Laemmli sample buffer.

Proteins were separated by polyacrylamide gel electrophoresis, transferred to a polyvinylidene difluoride (PVDF) membrane, and subjected to immunoblotting with the total OXPHOS antibody cocktail (ab110411). Then, the membranes were incubated with corresponding horseradish peroxidase-conjugated secondary antibody and visualized using an enhanced chemiluminescence system (ECL; Pierce, Thermo Fisher Scientific). After chemiluminescence reaction, the PVDF membranes were stained with Coomassie brilliant blue R250 to measure the total protein amount. The complexes I–V signal intensities were calculated by ImageJ software and normalized to total protein intensities.

Expression levels of complex I in HCC and normal tissues were additionally estimated using anti-NDUFA9 antibody that corresponds to NADH dehydrogenase 1*α* subcomplex 9 (SAB1100073). The samples were incubated and visualized as described above. Levels of NDUFA9 encoding protein were normalized to total protein content.

### 2.7. Citrate Synthase Activity

Activity of citrate synthase in tissue homogenates was measured as described by Srere [49]. Reactions were performed in 96-well plates containing 100 mM Tris-HCl pH 8.1, 0.3 mM AcCoA, 0.5 mM oxaloacetate, and 0.1 mM DTNB using FLUOstar Omega plate reader spectrophotometer (BMG Labtech).

### 2.8. Data Analysis

Data in the text, tables, and figures are presented as mean ± standard error (SEM). Results were analyzed by the Student *t*-test; *p* values <0.05 were considered statistically significant.

## 3. Results and Discussion

### 3.1. Respiratory Chain Analysis and Presence of Supercomplexes

Suppression of mitochondrial electron transport chain function is widespread in cancer, and this is closely connected to apoptosis resistance [50–54]. However, studies are often conducted on cell cultures and therefore little is known about respiratory chain (RC) function in clinical human breast and colorectal carcinomas in situ. To reveal possible disturbances, we conducted comparative quantitative analysis on the respiration rates for different RC complexes in permeabilized HBC and HCC and their adjacent normal tissue samples. Data for healthy breast tissue has been left out from most of the following calculations due to very low ADP-dependent oxygen consumption in this tissue type as it is not sufficient to assess inhibitory effects of antimycin A or rotenone or compare these results to other studied samples.

**Figure 1:**
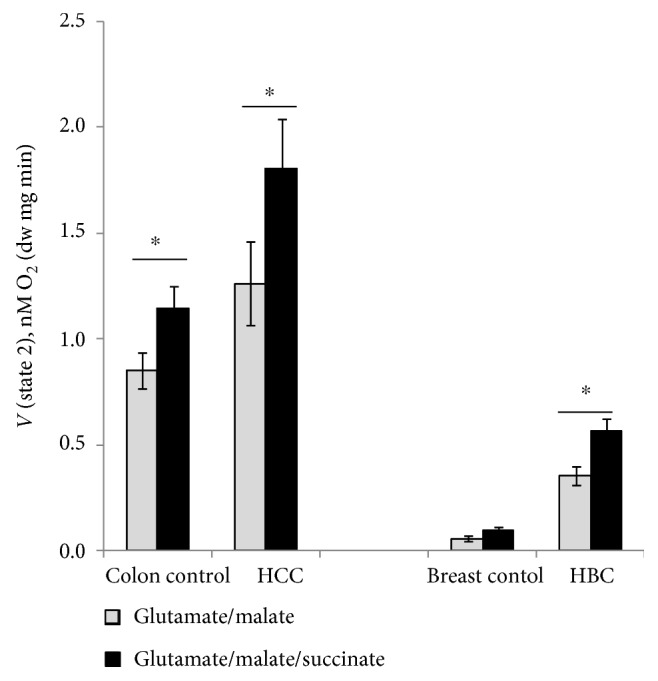
Assessment of state 2 respiration rates of the permeabilized HCC, HBC, and normal adjacent tissue samples in the presence of different combinations of respiratory substrates (5 mM glutamate, 2 mM malate, and 10 mM succinate). Bars are SEM, *n* = 8 for colon samples, and *n* = 12 for breast tissue samples, ^∗^*p* < 0.05.

Multiple substrate-inhibitor titration protocol was used for measuring respiratory capacities of different respiratory chain segments (Table 1) [30, 55]. All respiration rates corresponding to the activities of different RC complexes are increased in both investigated human cancers when compared to their adjacent normal tissue. The mean value of basal respiration (state 2, Vo) in skinned HCC samples is higher than that in normal tissue and depends on the used respiratory substrates. Specifically, in the presence of glutamate and malate, HCC and its control tissue fibers exhibit lower state 2 respiration rates than in the presence of glutamate, malate, and succinate; similar dependence was observed for the breast cancer samples (Figure 1). One possible reason for this difference can be succinate-dependent proton leak in tumor tissue [56–58]. Addition of 2 mM MgADP for studying complex I-based state 3 (in the presence of glutamate and malate without succinate) increased mitochondrial respiration rates in all tissue samples and following addition of complex I-specific inhibitor (rotenone) inhibited the respiration back to the initial state 2 levels (Table 1). Similarly, the function of complex II was quantified upon ADP-stimulated respiration in the presence of rotenone and succinate; at these conditions, the complex I activity is inhibited and apparent respiration rate originates from complex II. Complex III in both HBC and HCC was confirmed to be fully functional as an addition of antimycin A inhibited the electron flow from complex III to mitochondrial complex IV (COX) (Table 1). The activation of mitochondrial complex IV (addition of 5 mM ascorbate and 1 mM tetramethyl-p-phenylenediamine) resulted in a remarkable increase in the rate of O_2_ consumption in all examined samples, both cancerous and normal, but the increase was nearly two times higher in cancer tissue.

**Figure 2:**
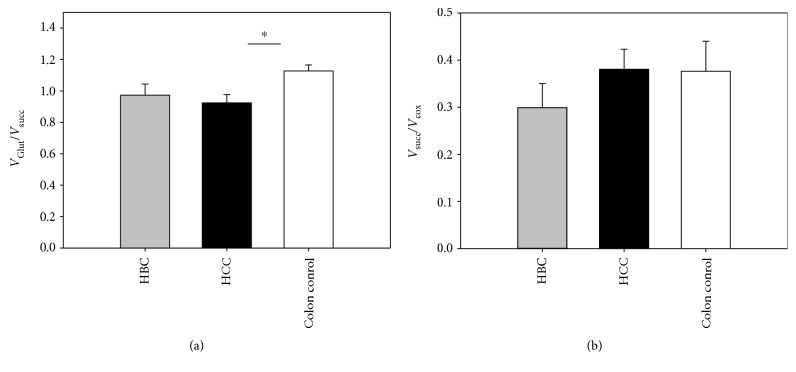
(a) Oxygraphic analysis of the functioning of complex I in skinned tissues from patients with HBC or HCC; here, *V*_Glut_/*V*_Succ_ is the ratio of ADP-stimulated respiration rate in the presence of 5 mM glutamate and 2 mM malate (activity of complex I) to ADP-stimulated respiration rate in the presence of 50 *μ*M rotenone and 10 mM succinate (activity of complex II). (b) *V*_Succ_/*V*_COX_ is the ratio of complex II respiration rate to complex IV respiration rate. Data shown as mean ± SEM; *n* = 7 for colon [28] and breast tissue samples [26], ^∗^*p* < 0.05.

**Figure 3:**
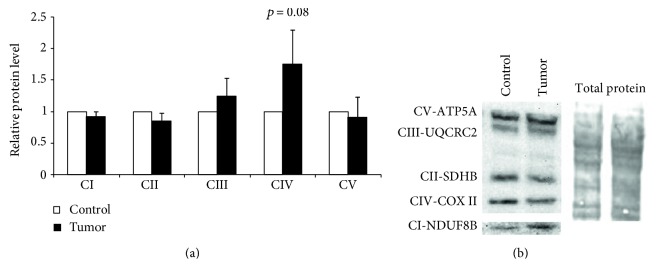
Quantitative analysis of the expression levels of the respiratory chain complexes in HCC and normal tissue samples (a) along with a representative Western blot image (b). Protein levels were normalized to total protein staining by Coomassie blue; data shown as mean ± SEM of 5 independent experiments.

Complex I deficiency is the hallmark of multiple mitochondrial diseases and is generally considered to be an intrinsic property of some cancers [58–63]. Indeed, our experiments confirm that development of HCC results in reduced *V*_Glut_/*V*_Succ_ ratio which indicates relative suppression of the complex I-dependent respiration [58]. Similar results have previously been described for gastric and ovarian cancer tissues but also in some cancer cell cultures [58, 63–66]. Deficiency of complex I in some tumors might be an early event causing an increase in mitochondrial biogenesis in an attempt to compensate for the reduction in OXPHOS function [63]. Computer modeling predicts that the mechanisms of this compensation can use multiple pathways like *β*-oxidation of fatty acids, mitochondrial folate metabolism, and others [67]. Our results showed that this suppression is pronounced on the functional level in HCC (Figure 2(a)), but to identify the changes on the protein expression level, we analyzed the RC complexes with total OXPHOS antibody cocktail (Figure 3). Based on this type of approach, the suppression of RC complex I was found to be absent if the results were normalized to total protein. The suppression of complex I in HCC was additionally studied by Western blot analysis with antibodies against only one complex I subunit—NDUFA9 (see Supplementary Fig 1 available online at https://doi.org/10.1155/2017/1372640). This result, however, confirmed the suppression of complex I. As seen from those experiments, analysis of RC, using the semiquantitative WB method, can be strongly dependent on experimental conditions: against what complex I subunit the antibodies were used and which normalization conditions are applied. Additionally, complex II in colon samples did not indicate possible suppression in that alternative pathway as differences in *V*_Succ_/*V*_COX_ ratios were not significant (Figure 2(b)) [68].

In contrast to HCC, mitochondrial respiration in HBC samples is not accompanied with suppression of complex I-dependent respiration (Figures 2(a) and 2(b)). Altogether, the relative complex I deficiency on the functional level in our oxygen consumption measurements is characteristic for HCC but not for HBC tissue.

Remarkable numbers of studies have shown that RC complexes can form protein assemblies (supercomplexes). These supramolecular structures provide kinetic advantage such as substrate channeling, increased efficiency in electron transport, prevention of destabilization, and degradation of respiratory enzyme complexes [21] and means to regulate ROS levels in the cell (most of the mitochondrial ROS originates from complexes I and III) [69] and hence, homeostasis. The RC complex I is considered to be the most important component in these assemblies, and it is a member of almost all known respirasomes [70–74]. In previous studies, complexes I, III, and IV were found to be assembled into supercomplexes in different configurations, but complex II was not confirmed to be a component of these RC supercomplexes and was assumed to move freely in the mitochondrial inner membrane [70, 75, 76]. Relative deficiency of the RC complex I on the functional level (as shown above) may be a result of changes in supercomplex composition as a part of malignant transformation.

In addition to RC supercomplexes, along with respirasomes, one more molecular transmembrane protein supercomplex (which is known as ATP synthasome; [77]) was identified as the component of the OXPHOS system. The ATP synthasome complex consists of ATP synthase, inorganic phosphate carrier, and adenine nucleotide translocator (ANT) [48]. The current model of mitochondrial interactosome (MI) considers the ATP synthasome and RC complexes together with voltage-dependent anion channel (VDAC) and mitochondrial creatine kinase (MtCK) as components of intracellular energetic units [22]. Even though MI is proven in striated muscles, the functional role of it, together with MtCK, in malignant samples remains controversial [78], but it indicates that both ATP synthasome and RC complexes can form even more complex functional structures.

**Figure 4:**
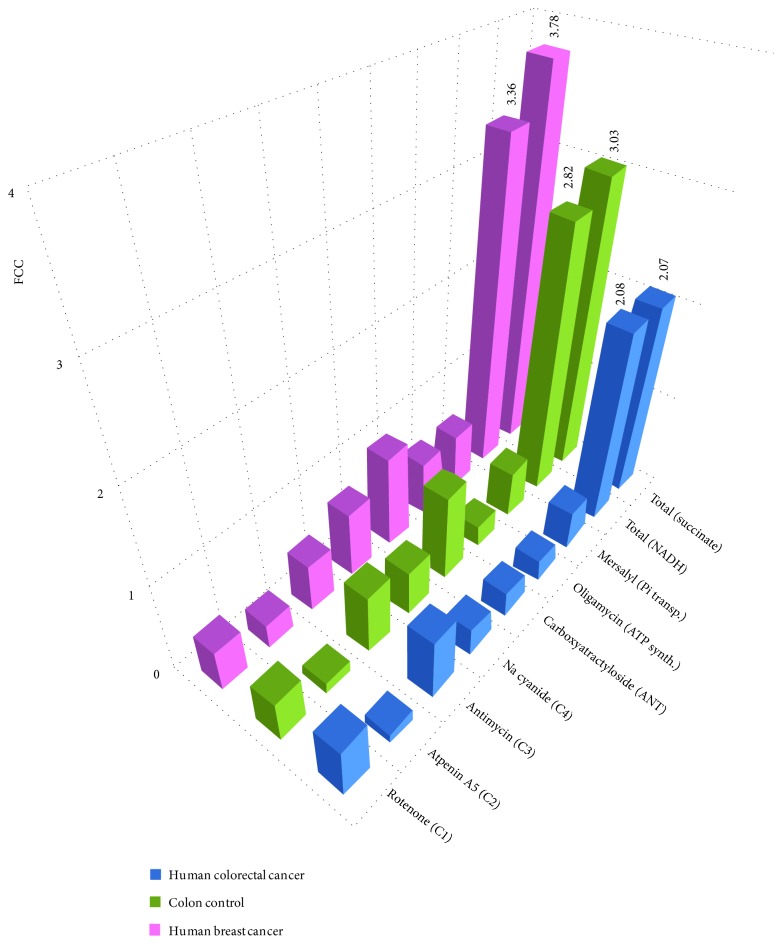
FCCs for ATP synthasome and RC complexes as determined by MCA. Two ways of electron transfer were examined: NADH-dependent and succinate-dependent electron transfers, and respective sums of FCCs are calculated as the last bars. Data for HBC is published before in [26], except for complex II with atpenin A5. Isolated mucosal tissue was used for colon control.

In addition to steady-state proteome studies, kinetic testing of metabolic fluxes using MCA can provide preliminary information about supramolecular organization in the energy transfer system and enables to quantify the flux exerted by the different RC and the ATP synthasome complexes [27, 28, 79]. MCA can discriminate between two prevailing models: the former model, based on the assumption that each enzyme can be rate controlling to a different extent, and a subsequent model, where whole metabolic pathway can behave as a single channel and inhibition of any of its components would give the same flux control [80]. Bianchi et al. proposed that both complexes I and III are highly rate controlling in NADH oxidation, suggesting the existence of functional association between these two complexes [80]. To confirm the formation of supercomplexes in HCC- and HBC-skinned samples using MCA, we investigated the flux control coefficients (FCCs) for the complexes involved in aerobic NADH oxidation (I, III, and IV), in succinate oxidation (II, III, and IV), and for components of the ATP synthasome. For this purpose, cancerous and normal tissue samples were titrated with increasing concentrations of specific inhibitors against all of the ATP synthasome and RC complexes.  Figure 4 summarizes the data analyzed in three different ways: by a graphical model [40, 41, 81], according to Small [82], and the Gellerich model [44]. The obtained FCC values did not depend on which exact method was used for calculations. The main problem in these calculations is high heterogeneity of the clinical material, which from the one hand originates from cancer molecular subtypes (e.g., Lumianal A/B, HER2 or triple negative in HBC; unknown subtypes in HCC) but on the other hand originates from heterogeneity of tumor cells within each tumor [14] or irregular stromal burden. Therefore, the obtained coefficient values do not only depend on which patients were included to the study, but the results may also depend on which particular tumor region was used from each patient sample. This can be considered as an inevitable part in analyzing clinical samples.

Previous work has shown that the main respiratory rate-controlling steps in HBC cells are complex IV (FCC = 0.74) and adenine nucleotide transporter (ANT, FCC = 1.02) [26]. Similar control distribution was not observed within HCC ATP synthasome complex as FCCs for ANT were found to be significantly lower when HCC was compared to the results of healthy colon mucosa (FCC = 0.284 for HCC and FCC = 0.970 for healthy colon). These results show that ANT exerts high flux control in healthy colon tissue (and in HBC), but ANT seems to lose its limiting role in HCC. Ramsay et al. believe that hexokinase-voltage-dependent anion channel-ANT complex, which spans across the outer and inner mitochondrial membranes, is critical in cancer cells as this complex is the link between glycolysis, oxidative phosphorylation, and mitochondrial-mediated apoptosis [83]. Therefore, the difference between HBC and HCC, in regard to ANT-exerted flux control, indicates to distinct difference in energy metabolism between these two tumor types (Table 2; Figure 4). In addition, HBC is showing equal FCCs for ATP synthase and inorganic phosphate transporter (Pi) in ATP synthasome, but this phenomenon is not characteristic neither for healthy colon mucosa nor for colorectal cancer. These alterations could be related to mitochondrial permeability transition pore (mtPTP) and apoptosis. Bernardi et al. studied the key regulatory features of the mtPTP [84–87], and the same group of authors has pointed to the fact that ANT can modulate the mtPTP, possibly through its effects on the surface potential, but it is not a mandatory component of this channel.

FCCs within the RC system in HBC do not differ significantly and the flux control is distributed almost uniformly throughout the different complexes (Table 2, Figure 4). Such condition is an indication of possible presence of protein supercomplexes (approximately equal values of FCCs for RC complexes I and III—0.46 versus 0.54, resp.). On the other hand, the flux distribution for normal colon tissue, when compared to HCC, showed slight difference for that for complex IV (FCC 0.50 versus 0.31), but flux control coefficients with close values were calculated for complex I (FCC 0.45 versus 0.56) and complex III (FCC 0.66 versus 0.68). Similarity in FCCs for complex I and complex III for both HCC and healthy colon tissue enables to propose that in healthy conditions, complex III is attached to complex I (possibly together with multiple copies of complex IV), but during carcinogenesis, the supercomplex assembly changes and even though complex I and complex III seem to stay linked, the participation of complex IV in this assembly becomes uncertain. Functional assembly of complexes I and III together with their rate-limiting roles will lead to sum of FCCs being greater than 1 [73] (see below).

Role of complex IV is multifaceted as three populations of it have previously been suggested: population assembled with complex I and complex III, population assembled with complex III alone and a non-interacting population [74]. Several data show that the absence of functional supercomplex assembly factor I (SCAF1) may be involved in distribution of complex IV [74, 75, 88]. As outlined in the review article by Enrìquez, total cell respiration (glucose, pyruvate, and glutamine as substrates) was significantly higher in cells lacking functional SCAF1 [74]. High total cell respiration was registered also for both cancer types described in this paper, but presence or absence of functional SCAF1 was not investigated.

The sums of the determined FCCs within cancerous and healthy sample groups were calculated to be in the range from 2.07 to 3.78. In theory, sum of FCCs in a linear system is 1 [5, 40, 42–44, 89, 90], but the value of it can increase if the system includes enzyme–enzyme interactions, direct channeling, and/or recycling within multienzyme complexes (i.e., system becomes nonlinear) [79, 80, 91, 92]. The higher sum of FCCs from our tests is not a result of diffusion restrictions because the concentration ranges for all of the inhibitors in various samples were similar and did not depend on the nature of the samples [26, 28, 47, 48].

The organization of RC complexes in the mitochondrial inner membrane has been an object of intense debate and it is not studied systematically in human normal or cancerous tissues. Given the known theoretical framework, our results confirm the plasticity model and agree with the data from Bianchi et al. [80], but the distribution of complex IV remains unclear—both random distribution and association into I-III-IV supercomplex can be possible. Large FCC for complex II is not characteristic neither for HBC, for HCC, nor for healthy colon tissue, and therefore, our kinetic studies confirmed previous findings that this complex is not a part of RC supercomplexes.

The question about the changes in the composition and stoichiometry of protein supercomplexes, which result from carcinogenesis, needs further studies, and in addition, as mitochondria have other additional roles in cellular metabolism, it can be presumed that changes in RC are also affecting cataplerotic processes sprouting from the mitochondria, but such link has not yet been studied yet.

### 3.2. ADP-Regulated Mitochondrial Respiration in HBC and HCC Fibers

**Figure 5:**
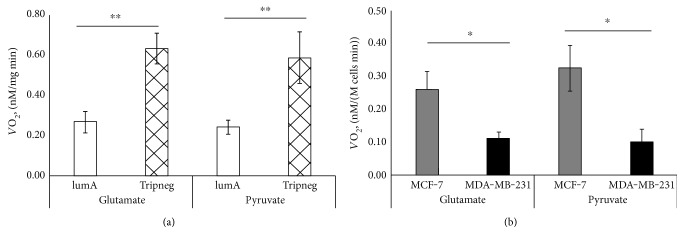
(a) Respiration rates for clinical samples of luminal-A and triple negative HBC subtypes in the presence of 5 mM glutamate or 5 mM pyruvate; *n* = 13/12 for luminal-A and *n* = 7/8 for triple negative subtypes, respectively. (b) Respiratory rates for luminal-A type MCF-7 and triple negative MDA-MB-231 cells in the presence of 5 mM glutamate or 5 mM pyruvate; *n* = 3 for each measurement; ^∗^*p* < 0.05, ^∗∗^*p* < 0.005.

Table 3 summarizes ADP-regulated mitochondrial respiration parameters determined for skinned tissue samples taken from both patient groups. Differences in the rates of maximal ADP-activated respiration (*V*_max_) in colon tissue samples are corresponding to the differences in the content of mitochondria in these cells (the amount of mitochondria in HCC is 50% higher than that in healthy control tissue [28] (supplementary Table 1)). Our previous experiments have shown that HBC tissue, too, contains an increased number of mitochondria in comparison to its adjacent normal tissue [27, 93] (supplementary Table 1). As indicated above, ADP-dependent respiration in healthy human breast tissue is absent. Breast samples contain lot of fat tissue, but low *V*_max_ values were evident even if clearly lobular/ductal structures were separated and tested. Low respiratory capacity can also be indicating to lowered metabolic activity in normal ductal/lobular tissue in older women (average age of HBC patients in this study was 63.4 years). In contrast to normal breast tissue, the colon control tissue samples have significantly higher respiration rates (Table 3). Specifically, respiratory capacity is higher in apparent mucosal/submucosal section of the normal colon tissue samples compared to that of the underlying smooth muscle part as we manually separated and tested these two layers in a selection of colon tissue samples (Figure 5(b)).

HBC arises from tissue with almost absent ADP-related respiration, but once formed, the mechanism of energy conversion seems to acquire a more complicated form and it can be associated with both increased mitochondrial biogenesis and interplay between cancer and stromal cells [26]. HBC can be classified into four clinically distinct and significant molecular subtypes: luminal-A, luminal-B, HER2 expressing, and triple negative. Clinically, luminal-A is considered the least and triple negative as the most aggressive subtype. Therefore, we expected to see clear differences when respiratory parameters of those two extreme subtypes were measured. Initially, respiration rates were analyzed in Luminal-A type MCF7 and triple negative MDA-MB-231 cell lines. When compared, respiration rates in presence of glutamate or pyruvate clearly showed that oxygen consumption in luminal-A subtype cells is remarkably higher (Figure 5(b)). But in contrast, the exact opposite was registered for the same parameters in clinical samples (Figure 5(a)) as the highest respiratory rates were registered for the most aggressive triple negative subtype. From the one hand, this contradicting result shows that cell cultures are not directly comparable to respective clinical counterparts and can lead to misguiding expectations. On the other hand, it proves that the role of OXPHOS becomes increasingly important in clinical samples as aggressiveness of the tumor increases, but it is not evident in the respective culture cells. In the present case, it is not a result of increased glucose availability in the growth medium, which could lead the cells to acquire glycolytic phenotype and explain the difference with clinical samples, because low-glucose media was used.

**Figure 6:**
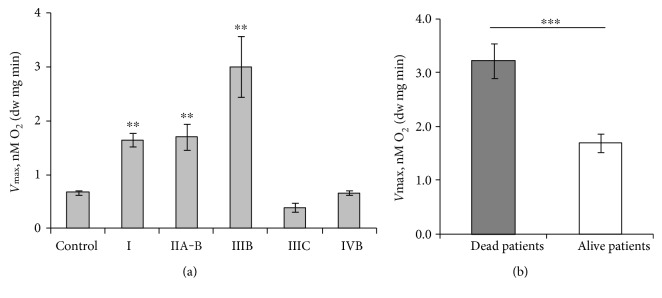
(a) Dependence of maximal rate of mitochondrial respiration (*V*_max_) compared with the HCC at different stages. Stage I was calculated as the mean of 13 patients, IIA, IIB - 13 patients, IIIB-4 patients, IIIC-3 patients and IVB-1 patient. Control colon tissue is obtained from 34 patients. Maximal respiration rate *V*_max_ is compared with that in control tissue. Bars are SEM; ^∗∗^*p* < 0.005. (b) *V*_max_ in HCC patients based on disease state in follow-up setting. Seven patients out of 32 are confirmed to have succumbed to HCC (*V*_max_ = 3.19 ± 0.34); 25 patients out of 32 stay in remission (*V*_max_ = 1.70 ± 0.17), ^∗∗∗^*p* < 0.001.

For HCC, which is without distinct clinical subtypes, we compared disease stage to average *V*_max_ value for that stage (Figure 6(a)). Even though increase in *V*_max_ in initial stages can be calculated in comparison to control sample, the decrease in *V*_max_ for stages IIIC and IVB does not fit this increase in dependence. The disease stage at diagnosis itself is not a valid marker of aggressiveness and therefore such plotting can be debated. Therefore, we gathered initial longitudinal data on patient progression in our HCC cohort and confirmed that 7 out of 32 eligible patients had died (median follow-up time 47.3 ± 4.9 months). *V*_max_ values in patients that succumbed to the disease were significantly higher than that in the currently not progressed group (Figure 6(b)). As was shown for HBC above, higher respiratory capacity was registered for the most aggressive triple negative subgroup. Therefore, it can be argued based on similarity that higher tumor respiratory parameters in the dead HCC patients were indicating to more aggressive disease. In addition, lower than expected respiratory rate in some triple negative tumors can therefore indicate that given patient, when compared to the average in the triple negative subgroup, has less aggressive disease than could be expected. To confirm this in larger cohorts and relate aggressiveness in HCC and HBC to *V*_max_ value, additional longitudinal studies are necessary.

We next measured apparent Michaelis–Menten constants (*K*_m_) for ADP to characterize the affinity of mitochondria for exogenous ADP (i.e., permeability of mitochondrial outer membrane). Corresponding *K*_m_ values for permeabilized tumor and nontumorous tissues were determined from titration experiments using exogenously added ADP. The obtained data were plotted as rates of O_2_ consumption versus ADP concentration and apparent *K*_m_ values were calculated from these plots by nonlinear regression equation. Healthy colon tissue displayed low affinity for ADP (*K*_m_ = 256 ± 3 *μ*M), whereas that in HCC is significantly higher (*K*_m_ = 93.6 ± 7.7 *μ*M) [38]. The *K*_m_ (ADP) value for HBC tissue samples (*K*_m_ = 114.8 ± 13.6 *μ*M) was similar to that for HCC [26].

According to the classical studies by Chance and Williams [94, 95] and the data of many other investigators [29, 30, 37], the apparent *K*_m_ value for ADP for isolated mitochondria is low, about 15 *μ*M, but the observed apparent *K*_m_ values in our study for permeabilized clinical HBC and HCC samples were 6–8 times higher than this value (Table 3). Our previous studies have shown that sensitivity of the mitochondrial respiration for exogenous ADP for permeabilized NB HL-1 cells is also high as the apparent *K*_m_ equaled to 25 ± 4 *μ*M and was similar to that of isolated heart mitochondria [34, 96]. The similar low apparent *K*_m_ values were also registered for undifferentiated and differentiated neuroblastoma culture cells, where the corresponding *K*_m_ for ADP were measured as 20.3 ± 1.4 *μ*M and 19.4 ± 3.2 *μ*M, respectively [97]. The registered difference between culture cells and clinical samples, despite the used preparation method, again indicates to differences present in these two sample groups.

**Figure 7:**
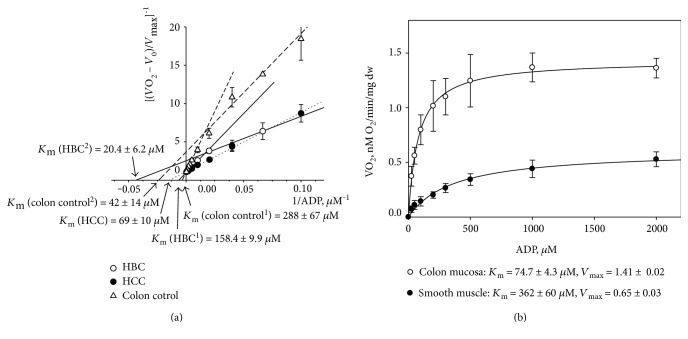
(a) Dependences of normalized respiration rate values for HCC (dotted line), HBC (solid line), and healthy colon tissue samples (dashed line); double reciprocal Lineweaver–Burk plots. Samples from 32 patients with breast cancer and 10 patients with colorectal cancer were examined. (b) ADP-dependent respiration in healthy colon mucosa and smooth muscle tissue samples (Michaelis–Menten curve, *n* = 8). Here, Vo and *V*_max_ are rates of basal and maximal ADP-activated respiration, respectively.

We treated permeabilized samples with incremental concentrations of ADP and the measured O_2_ consumption rates (normalized to *V*_max_) were analyzed against respective ADP concentration values as double reciprocal Lineweaver–Burk plots (Figures 7(a) and 7(b)) [29].  Figure 7(a) shows the results of the Lineweaver–Burk treatment of the experimental data linked with ADP-regulated mitochondrial respiration in skinned fibers of HCC, healthy colon, and HBC. Corresponding *V*_max_ and *K*_m_ values were calculated from the linearization approach. Saks and colleagues have previously shown that the presence of biphasic respiration regulation on the graph curve indicates the existence of two populations of mitochondria with different affinities for ADP [29]. Our results indicated such differences in colon control and HBC samples. Specifically, monophasic regulation of mitochondrial respiration is apparent in HCC tissue, but in healthy colon tissue, two populations of mitochondria with very different properties were found (Figure 7(a)). One population of mitochondria is characterized with lower *K*_m_ (42 ± 14 *μ*M), whereas the apparent *K*_m_ (ADP) value for the second mitochondrial population is nearly seven times higher (288 ± 67 *μ*M). We thereafter again separated mucosal and smooth muscle parts from the colon samples before additional *K*_m_ measurements to characterize their isolated contributions. Apparent *K*_m_ value for mucosal part was measured to be 74.7 ± 4.3 *μ*M and the same value for colon smooth muscle tissues was found to be 362 ± 60 *μ*M (Figure 7(b)). Therefore, results after separation explain the results from the initial experiment where the entire colon wall was analyzed and two separate groups of mitochondria were discovered. Additionally, we could also distinguish two differently regulated types of mitochondria in HBC samples: one with apparent *K*_m_ value for MgADP of 20.4 ± 6.2 *μ*M, but the same for the second mitochondrial population was nearly ten times higher, 158.5 ± 9.9 *μ*M (Figure 4(a)). The phenomenon shown in Figure 7(a) can be associated, on the one hand, simply with elevated stromal content (in such case, similar results should have been also registered for HCC), but on the other hand, with possible two-compartment tumor metabolism in HBC, what states that tumor cells function as metabolic parasites and extract energy from supporting host cells such as fibroblasts [98–103]. In such case, the stromal part of the HBC samples can be characterized with glycolytic metabolism representing the low *K*_m_ value due to high levels of autophagy, mitophagy, glycolysis, and lipolysis, while cancer cells have high mitochondrial mass, OXPHOS, and *β*-oxidation activity, which is represented by the mitochondrial population with the high *K*_m_ (ADP) values. From the given comparison between HBC and HCC, the two subpopulations of mitochondria are specific only to HBC samples (confirmed in 32 cases out of the total 34) but not to HCC samples, and it indicates that tumor formation leads to distinct changes, which is related to the tissue type the tumor originates from.

Altogether, these results indicate the remarkable differences in the regulation of mitochondrial outer membrane (MOM) permeability between cultured tumor cells and clinical material (including between different tumor types and even between patients). Even further, the results can be contradictory as registered for respiration parameters. It can be estimated, based on the results from our lab, that low *K*_m_ value for ADP can be a common characteristic for cancer cells grown in culture, but in in vivo tumor samples, the regulation of MOM permeability is more complicated and probably related to interplay between energy transfer pathways and changes in the phosphorylation state of VDAC channels [32, 104–107] and also with modulation of cytoskeleton or membrane potential as a result of tumor formation.

## 4. Concluding Remarks

**Figure 8:**
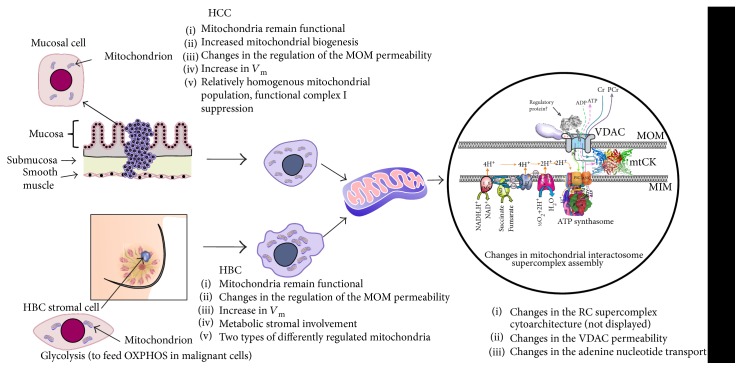
Mitochondrial alterations in HCC and HBC tissue cells. Mitochondrial interactosome is a large supercomplex consisting of ATP synthasome, VDAC, mitochondrial kinases like adenylate kinase, hexokinase or mitochondrial creatine kinase (MtCK), and respiratory chain (super)complexes. Here, the octameric MtCK characteristic is shown for the striated muscles and also as the possible component of the MI in the healthy colon [108–110]. The complex of VDAC together with other proteins controls the exchange of adenine nucleotides and regulates energy fluxes between mitochondrial and cytosolic compartments. Changes in the structure and function of MI are the important parts of cancer mitochondrial metabolism.

To understand the energy metabolism of tumors, it is necessary to detect bioenergetic fingerprints of each individual tumor type. Our results confirmed that respiratory capacity is preserved in both HBC and HCC as these both demonstrated substantial rates of oxidative phosphorylation, which contradicts with earlier widespread understanding that the metabolism of human breast and colorectal carcinomas is prevalently glycolytic. Studies on cell lines up to now have led to many lifesaving technologies and treatments in humans, but the scientific level might be nearing the end of readily transferrable results between the cell model and human physiology. Our results indicated that apparent glycolytic nature of some breast cancer types could be expected based on cell cultures, but this presumption was in sharp conflict when culture cell results were compared with these from respective clinical samples. In addition, when compared to their healthy adjacent tissue, both clinical cancer types showed increased respiratory capacity. Despite the increased respiratory capacity in HCC, relative deficiency of complex I was registered for it on the functional level Western blot analysis was not sufficient to confirm this deficiency on the protein level as two different antibody approaches gave conflicting results, but this result proves the necessity to measure pathways also on the functional level whenever possible to compare the function to steady-state markers like presence or abundance of certain enzymes. Our experiments indicate that the respiratory chain and ATP synthasome can form macromolecular assemblies (supercomplexes) with reorganized composition and/or stoichiometry while the changes are specific for different tumor types. This is in good agreement with recent studies from other laboratories [25] and the current work shows that equal results can be obtained using kinetic methods, but additional studies are warranted to include results from protein level studies using the blue native gel electrophoresis (BNGE) technique. Our *K*_m_ measurements confirmed that two populations of mitochondria registered in healthy colon tissue can be categorized as different layers of the colon wall, but in HBC, the subgroups can be linked to two-compartment metabolism where tumor acts as a metabolic parasite on normal stromal cells. Mitochondria of HCC are homogenous in terms of regulation of the mitochondrial outer membrane permeability and MCA (Figure 8).

Mitochondria are not only the centers of cellular energy conversion but are also the important part in biosynthetic metabolism and apoptosis. Therefore, direct detection of profound changes in the ATP synthasome components and in the architecture of the respiratory chain complexes, as shown in the current work, can support development of new predictive models or therapies.

## Supplementary Material

Supplementary Table 1. Activity of citrate synthase as marker of mitochondrial mass [105], in human breast and colorectal cancers as well as in corresponding normal tissues. Enzymatic activity measured as mU/mg protein, mean ± SE. Supplementary Fig. 1. Quantification of Complex I subunit NDUFA9 expression levels using Western blot (WB) and anti-NDUFA9 antibody in HCC and healthy colon tissue samples (A); respective WB image of NDUFA9 protein levels (B). Levels of NDUFA9 were normalized to total protein content quantified by coomassie blue staining. Data are represented as mean values ± SEM from 4 independent experiments; ∗ p<0.05.

## Figures and Tables

**Table 1:** Characterization of respiratory parameters of permeabilized tissue samples derived from patients with breast or colorectal cancer.

Parameters	HBC patients, *n* = 7 [26]		HCC patients, *n* = 7 [28]	
	Tumor	Control	Tumor	Control
Vo	0.294 ± 0.024	0.004 ± 0.007	1.06 ± 0.14	0.82 ± 0.15
V_ADP_	0.71 ± 0.06	0.055 ± 0.004	2.02 ± 0.21	1.39 ± 0.21
Vrot	0.34 ± 0.04	0.070 ± 0.015	0.91 ± 0.11	0.85 ± 0.14
*V*_Succ_	0.74 ± 0.10	0.076 ± 0.008	2.22 ± 0.26	1.33 ± 0.18
VANM	0.38 ± 0.04	0.071 ± 0.018	1.04 ± 0.09	0.69 ± 0.07
*V*_COX_	2.36 ± 0.33	1.23 ± 0.18	6.59 ± 0.71	3.84 ± 0.58

Note: here, each data point is the mean ± SEM of respiratory values. Vo: basal respiration without ADP or ATP; V_ADP_: ADP-stimulated respiration (final concentration 2 mM) in the presence of 5 mM glutamate and 2 mM malate (indicating the function of the respiratory chain complex I); Vrot: rates of respiration after addition of 50 *μ*M rotenone (an inhibitor of complex I); *V*_Succ_: ADP-stimulated respiration in the presence of rotenone and 10 mM succinate (to estimate the function of complex II); VANM: rates of respiration after addition 10 *μ*M antimycin-A (an inhibitor of complex III); *V*_COX_: rates of O_2_ consumption in the presence of complex IV substrates (5 mM ascorbate jointly with 1 mM tetramethyl-p-phenylenediamine).

**Table 2:** FCCs for different components in mitochondrial Interactosome and ranges of the concentrations of inhibitors.

MI component	Inhibitor	Range of inhibitor concentration		FCC	
			HCC	Control colon tissue (mucosa)	HBC
Complex I	Rotenone	1–100 nM	0.56	0.45	0.46^∗^
Complex II	Atpenin A5	0.1–6 *μ*M	0.12	0.13	0.28
Complex III	Antimycin	1–200 nM	0.68	0.66	0.54^∗^
Complex IV	Na cyanide	0.1–40 *μ*M	0.31	0.50	0.74^∗^
ANT	Carboxyatractyloside	1–200 nM	0.28	0.97	1.02^∗^
ATP synthase	Oligomycin	1–600 nM	0.25	0.24	0.61^∗^
Pi transporter	Mersalyl	1–200 *μ*M	0.43	0.53	0.60^∗^
**Sum 1, 3–7**	**Total (NADH)**		**2.08**	**2.82**	**3.36**
**Sum 2–7**	**Total (succinate)**		**2.07**	**3.03**	**3.78**

Note: ^∗^from [26].

**Table 3:** Apparent *K*_m_ (^app^*K*_m_) and maximal rate of respiration (*V*_max_) values for ADP-dependent respiration calculated for HBC, HCC and their adjacent healthy tissue samples.

Tissues	^app^ *K*_m_, *μ*M ± SEM	*V*_max_ ± SEM
Human breast cancer tissue	114.8 ± 13.6^∗^	1.09 ± 0.04^∗^
Healthy adjacent breast control tissue	—	0.02 ± 0.01^∗^
Human colorectal cancer tissue	93.6 ± 7.7^∗∗^	2.41 ± 0.32
Healthy adjacent colon control tissue	256^∗∗^ ± 34	0.71 ± 0.23

Note: ^∗^from [26] and ^∗∗^ [38]; *V*_max_ values are presented as nM O_2_/min/mg dry tissue weight without proton leak rates. These *K*_m_ and *V*_max_ values for ADP were determined from corresponding titration curves by fitting experimental data to non-linear regression equation according to a Michaelis–Menten model. 35 patients used for analysis of HBC and 35 for HCC.

## Abbreviations

ANT: Adenine nucleotide translocator
CAT: Carboxyatractyloside
COX: Cytochrome c oxidase
FCC: Flux control coefficient
HBC: Human breast cancer
HCC: Human colorectal cancer
HK: Hexokinase
MCA: Metabolic control analysis
MI: Mitochondrial interactosome
MOM: Mitochondrial outer membrane
MtCK: Mitochondrial creatine kinase
mtPTP: Mitochondrial permeability transition pore
OXPHOS: Oxidative phosphorylation
RC: Respiratory chain
SCAF1: Supercomplex assembly factor I
TME: Tumor microenvironment
VDAC: Voltage-dependent anion channel.

## Additional Points

*Highlights*. (1) Relative complex I functional deficiency is characteristic for HCC but not for HBC. (2) HBC respiratory capacity severely higher than in adjacent normal breast tissue. (3) Complexes I and III expectedly assembled in both tumorous and normal tissues. (4) *K*_m_ for ADP shows distinct differences between cell cultures and clinical samples. (5) Two distinct mitochondrial populations present in HBC but not in HCC.
