# Supplementary material for: Mitochondrial Respiration in Human Colorectal and Breast Cancer Clinical Material Is Regulated Differently
Source: Oxid Med Cell Longev. 2017 Jul 11;2017:1372640. doi: 10.1155/2017/1372640 (PMC5525093; doi:10.1155/2017/1372640)
Supplement: Supplementary file 1 — Supplementary Table 1. Activity of citrate synthase as marker of mitochondrial mass [105], in human breast and colorectal cancers as well as in corresponding normal tissues. Enzymatic activity measured as mU/mg protein, mean ± SE. Supplementary Fig. 1. Quantification of Complex I subunit NDUFA9 expression levels using Western blot (WB) and anti-NDUFA9 antibody in HCC and healthy colon tissue samples (A); respective WB image of NDUFA9 protein levels (B). Levels of NDUFA9 were normalized to total protein content quantified by coomassie blue staining. Data are represented as mean values ± SEM from 4 independent experiments; ∗ p<0.05. [file 1372640.f1.pdf]

Supplementary Table 1. Activity of citrate synthase as marker of mitochondrial mass [105], in human breast and colorectal cancers as well as in corresponding normal tissues. Enzymatic activity measured as mU/mg protein, mean ± SE.

**Samples**	**Enzymatic activity**	***p*-values***
Human breast cancer	14.55 ± 2.59; n = 9	< 0.005
Healthy breast tissue	4.09 ± 1.15; n = 9	
Human colorectal cancer	83 ± 19; n = 6	< 0.05
Healthy colon tissue	46 ± 6; n = 6	

Notes: * significant difference between malignant *vs.* adjacent healthy tissue samples.

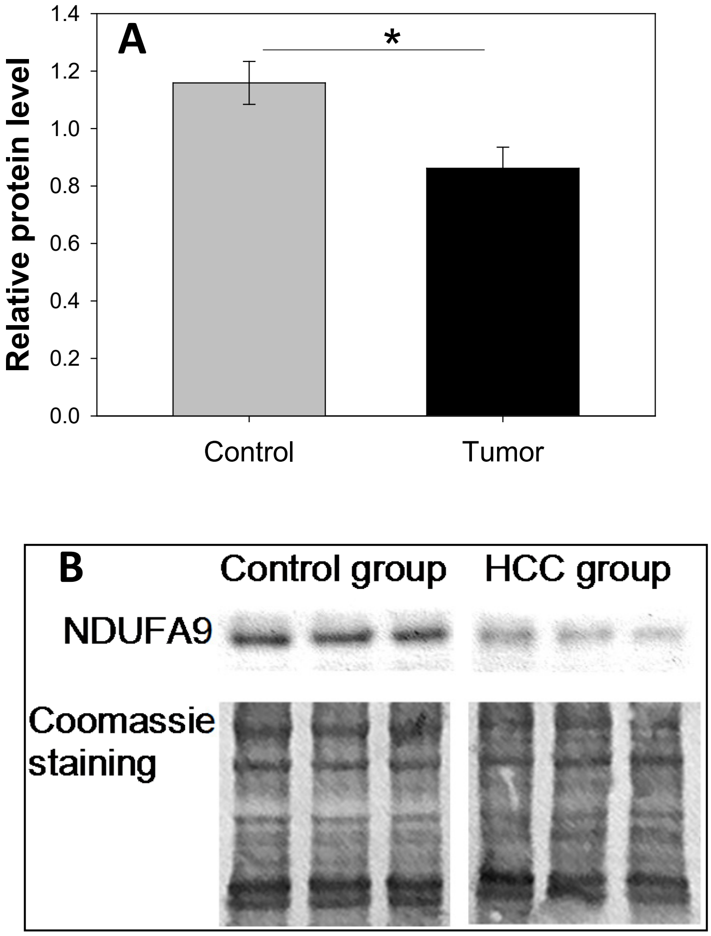

Supplementary Fig. 1. Quantification of Complex I subunit NDUFA9 expression levels using Western blot (WB) and anti-NDUFA9 antibody in HCC and healthy colon tissue samples (**A**); respective WB image of NDUFA9 protein levels (**B**). Levels of NDUFA9 were normalized to total protein content quantified by coomassie blue staining. Data are represented as mean values ± SEM from 4 independent experiments; * $p<0.05$.
